# Flexible Gas Sensors Employing Octahedral Indium Oxide Films

**DOI:** 10.3390/s18040999

**Published:** 2018-03-28

**Authors:** Miriam Alvarado, Èric Navarrete, Alfonso Romero, José Luis Ramírez, Eduard Llobet

**Affiliations:** MINOS-EMaS, Universitat Rovira i Virgili, Avda. Països Catalans, 26, 43007 Tarragona, Spain; miriam.alvarado@urv.cat (M.A.); eric.navarrete@urv.cat (È.N.); alfonso.romero@urv.cat (A.R.); joseluis.ramirez@urv.cat (J.L.R.)

**Keywords:** indium oxide, flexible substrates, gas sensors, nitrogen dioxide

## Abstract

Indium oxide octahedral nanopowders were obtained from an ionic precursor compound after an oxidation process conducted under a low-oxygen atmosphere. This method was found to produce contamination-free indium oxide nanomaterial with very similar morphological and crystalline properties to the one produced by vapor-phase transport, but at significantly lower temperatures and higher yield. The as-synthesized indium oxide was mixed to an organic vehicle and microdrop deposited to form a film bridging the interdigitated silver electrodes patterned on top of a flexible, polyimide (Kapton^®^), substrate. The gas sensing properties of the flexible chemoresistors towards ammonia vapors, hydrogen, and nitrogen dioxide were investigated. It was found that these sensors were remarkably sensitive to nitrogen dioxide at a low operating temperature of 150 °C. These results are consistent with the performance of vapor-phase transport synthesized indium oxide octahedra sensors on rigid, ceramic substrates. Therefore, the results presented here pave the way for the mass production of inexpensive gas sensors onto flexible substrates via additive manufacturing.

## 1. Introduction

Indium oxide (In_2_O_3_) belongs to the family of metal oxides, such as tin oxide, tungsten trioxide, and zinc oxide, which have been reported as promising materials for gas sensing applications. In_2_O_3_ is an n-type semiconductor with a band gap of 2.7 eV. This material has been widely studied for gas sensing. Different shapes and material morphologies (nanofilms, nanowires, nanorods, octahedra) [1,2] have been already tested. Nanostructured In_2_O_3_ has been reported mainly for detecting nitrogen dioxide (NO_2_), hydrogen (H_2_), and ammonia (NH_3_) [3,4,5]. Sol-gel, chemical vapor deposition, laser ablation, or vapor phase deposition have been employed to grow In_2_O_3_ nanomaterials. Recently, we reported the growth of In_2_O_3_ nano-octahedra employing vapor-phase transport (VPT) at 1000 °C under an Ar atmosphere [6]. In_2_O_3_ octahedra obtained by VPT possess sharp edges and vertices, and were found to be highly sensitive and remarkably selective to NO_2_ when operated at moderate temperatures (i.e., 130 °C). However this growth method has the disadvantage—besides the high operating temperatures needed—of producing small quantities of the nanomaterial. Here we report an alternative process for obtaining high quantities of octahedral In_2_O_3_ at a significantly lower temperature of 500 °C. Additionally, this growth method is conducted without using any solvent or carrier gases. 

Chemoresistive metal oxide sensors require simple transducers, basically consisting of an inert substrate comprising a pair of interdigitated electrodes onto which the gas sensitive film is deposited or printed and a heating resistor. Ideally, substrates are required to withstand high temperatures, show chemical inertness, and behave as barriers to gases and moisture [7]. Ceramic (e.g., alumina) and silicon based substrates have been, by far, the most used substrates for developing gas sensors [8,9]. However, the replacement of these conventional, rigid materials by flexible ones would bring some advantages such as lowering cost and heat losses and widening the field of applications to wearable or disposable sensing systems. Nowadays, flexible devices—such as photodetectors, light emitting diodes, pressure sensors, artificial electronic skin, and biomedical sensors—have been fabricated [10]. The most used materials for implementing flexible substrates have been plastic films, metal foils, and fibrous materials (including paper and textiles) [11]. Among polymers, polyimide (PI), polyether ether ketone (PEEK), polyethylene terephthalate (PET), and polyethylene naphthalate (PEN) have been determined to be suitable materials for the fabrication of flexible substrates and devices [12,13]. Moreover, all these polymeric materials have been often selected to fabricate flexible devices for sensing applications [14]. Among the above mentioned polymeric materials, PI exhibits temperature resistance—essential for metal-oxide deposition and sensor operation—and allows to print circuit tracks. Gas sensors on PI foil have been reported and results were comparable to those obtained on standard silicon micromachined transducers [7]. Additionally, it has been demonstrated that In_2_O_3_ gas sensors on polymeric transducers behave similarly to those employing ceramic transducers [15]. In this paper, a low-cost, off-the-shelf adhesive polyimide has been used as a substrate to fabricate metal oxide sensors, employing In_2_O_3_ as a sensing layer. These new results represent a significant advancement in comparison with our previously reported prototypes, as far as fabrication and deposition processes have been improved. Namely, the material synthesis process has been optimized and the coating procedure has been readapted in order to increase reproducibility. Those sensors were tested for three different gases. These devices bring good openings for developing flexible sensors employing metal oxides in the near future.

## 2. Materials and Methods

### 2.1. Material Preparation

In_2_O_3_ was obtained by employing commercially-available indium chloride (InCl_3_, Sigma Aldrich, St. Louis, MO, USA, 99.8% purity) via an oxidation process. This precursor is stored at 0 °C because indium chloride starts sublimating at 5 °C. In a typical synthesis experiment, 40 mg of InCl_3_ were weighed and put in a ceramic crucible, which was immediately placed inside a muffle. Three tests were carried out to determine the best oxidizing temperature, setting the muffle at 400 °C, 500 °C, and 600 °C. Generally, in the oxidation process, a current of pure, dry air is used to increase the availability of oxygen species [11]. In our experiments, the oxidation process was conducted without employing an air flow in order to obtain a crystal oxide structure under low oxygen concentration. Once the crucible was placed inside the muffle, its temperature was increased progressively at a 5 °C·min^−1^ rate, until it reached the oxidizing temperature (i.e., 400 °C, 500 °C, or 600 °C). Once reached, the oxidation temperature was kept for 120 min. Finally, the material was left inside the muffle to naturally cool down to room temperature. The success in the oxidation process could be checked by the naked eye, given the change in color experienced from white InCl_3_ to light yellow In_2_O_3_. Before any further processing, the obtained materials were placed inside an agate mortar and mashed softly until they turned into a homogenous yellowish dust. 

### 2.2. Sensor Fabrication

Transducers comprise two interdigitated (IDE) silver electrodes over a polymeric substrate. The width of the IDE fingers is 300 µm and the electrode gap was 300 µm. The surface of the electrode area was 2.4 × 5.7 mm. As a substrate, a commercially-available, highly-resistant, adhesive-coated polyimide foil (Kapton^®^ 25 µm) was used. For the fabrication of the electrodes, an additional layer of adhesive-coated polyimide was used as a shadow mask. Over the external layer, a finger-shape electrode pattern was cut by a CO_2_ laser. Afterwards, the surface was wiped off with a towel moistened with ethanol (ethanol 96% pure, Sigma Aldrich). Using a pair of tweezers, the material belonging to the pattern was removed, thus obtaining a negative mask. Silver (Ag) ink (DuPont^®^ 5064H, silver conductor, Wilmington, DE, USA) was stenciled with a spatula onto the polyimide. Then, the ink was dried inside an oven for 20 min at 130 °C. Subsequently, the shadow mask was peeled-off obtaining two IDE deposited on the polymeric substrate.

In order to obtain a sensing layer of In_2_O_3_ octahedra, a microdrop coating technique was employed to coat the electrode area of the transducer. To implement such a technique, 20 mg of nanomaterial were placed inside an Eppendorf and mixed with 0.5 mL of 1,2-propanediol to obtain a suspension. The suspension was agitated to ensure a homogeneous suspension and using a micropipette, 1 µL droplet was deposited on top of the electrode area. Subsequently, the sensor was placed inside an oven at 150 °C to enable solvent evaporation. This process was repeated up to four times for each device, until film resistance ranged between 3 and 6 MΩ at room temperature. 

## 3. Results

### 3.1. Material Characterization

**Figure 1:**
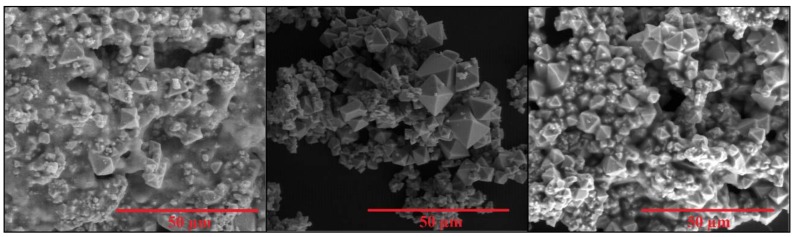
E-SEM micrographs showing the evolution of the morphology of indium oxide materials as a function of the temperature employed during the synthesis. (**Left**), (**middle**), and (**right**) panels correspond to an oxidation step conducted at 400 °C, 500 °C, and 600 °C, respectively.

The morphology—chemical composition and crystalline phase—of the different In_2_O_3_ materials obtained were characterized using environmental scanning electron microscopy (E-SEM), energy-dispersive X-ray spectroscopy (EDX) and X-ray diffraction (XRD), respectively. An E-SEM (FEI QUANTA600, Hillsboro, OR, USA) coupled to an EDX (Oxford Instruments, Abingdon, UK) were employed to study the structure and composition of materials. Figure 1 summarizes E-SEM results showing the morphologies of the In_2_O_3_ nanomaterials for the different synthesis temperatures employed.

When the oxidation is conducted at 400 °C the resulting indium oxide contains a few octahedra, however, it mainly consists of particles of irregular shape and amorphous material. In contrast, when the oxidation was conducted at 500 °C, indium oxide appears in the form of octahedra with well-defined surfaces and sharp edges and vertices. At 600 °C even though octahedra are still present, many are fused together and a significant amount of particles re-appear. These studies reveal that when the synthesis is conducted at 500 °C, the octahedral morphology is improved.

EDX results (supporting information) indicate the presence of In, O, and Cl for samples oxidized at 400 °C. In the literature it has been reported the poisoning effect of chlorine ions. Several studies have been conducted to understand the effect of such ions; when there are either on the material surface or included in the metal oxide structure. Chlorine ions can interact with different types of metal oxides in a way that leads to different effects such as their corrosion or breakage, reducing their performance and lifetime [16]. Chlorine contamination is indicative that the precursor has not been fully oxidized and removed by the process conducted at 400 °C. Therefore, in an attempt to eliminate the Cl surplus, different oxidizing temperatures have been tested. In contrast, for samples oxidized at 500 °C and 600 °C only In and O are detected, indicating that Cl is completely removed or is present at concentrations that are under the limit of detection of the EDX technique. 

Considering the morphology and composition results revealed by E-SEM and EDX, the optimal oxidizing temperature was 500 °C. Once the optimal growth temperature is known, a yield study was carried out to determine the efficiency of such a type of growth, 20.5 mg In_2_O_3_ were obtained employing 50 mg of InCl_3_ precursor, resulting in an excellent global yield of 65%. Most of these losses occur during the transfer of the material from the ceramic crucible were it is grown to the agate mortar and from there to the Eppendorf where a suspension of indium oxide octahedra is prepared. 

Considering the microdrop coating method employed for depositing the gas-sensitive material onto transducers, 20 mg of nanomaterial are enough for producing more than 100 sensors. Additionally, this high-yield synthesis process can be easily scaled up and is simpler to implement than VPT. Unlike VPT, the method employed here is run at 500 °C in a standard muffle under atmospheric conditions, avoiding the use of a tubular quartz furnace under atmospheres of controlled gas flows. Therefore, it represents a good option for the inexpensive, mass production of materials and sensors.

**Figure 2:**
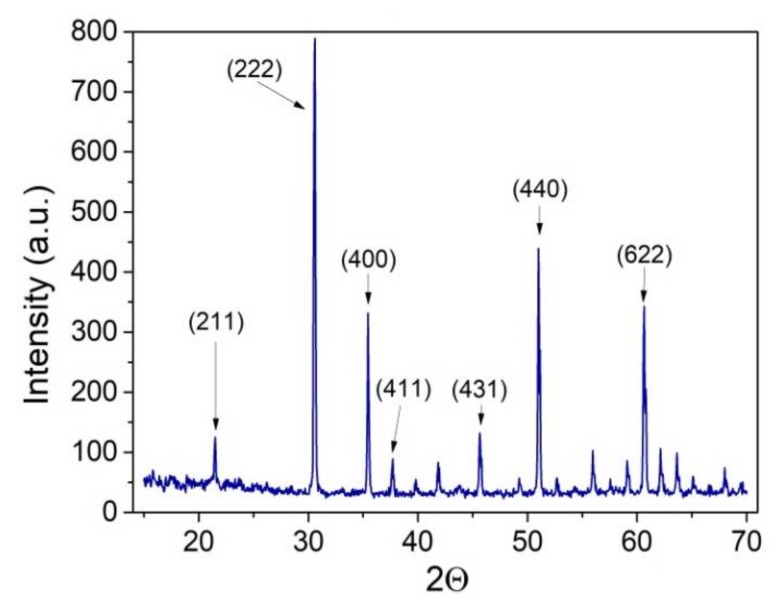
Typical XRD pattern of an In_2_O_3_ sample synthesized at 500 °C.

The crystalline phase of samples grown at the optimal temperature of 500 °C was studied by XRD. These results are presented in Figure 2 where the diffraction pattern correlates with the typical cubic structure of In_2_O_3_ corresponding to space group Ia-3 with an a° = 10.12 Å. The In_2_O_3_ displays a bixbyite structure belonging to the space group 206 mentioned before. This space group corresponds to a body-centered cubic structure formed by 40 atoms in the primitive cell. Two non-equivalent lattice positions are occupied by indium atoms being surrounded by oxygen in trigonal and octahedral prismatic coordination, which results finally in the octahedral shape of the nanostructures observed by E-SEM. 

All the peaks found in the spectrum correspond to each one of the faces displayed by the material with lower or higher intensity, fitting with the corresponding JCPDS card no. 01-071-2194. The most representative peaks appear labelled in Figure 2. None of the peaks present could be attributed to impurities, confirming the purity of the material synthesized [17]. The results of the XRD analysis for the sample synthesized at 500 °C match those reported previously for In_2_O_3_ octahedra synthesized at 1000 °C via the VPT method [6], which indicates that the crystalline quality of the samples discussed in this paper is very similar to the one reported before.

**Figure 3:**
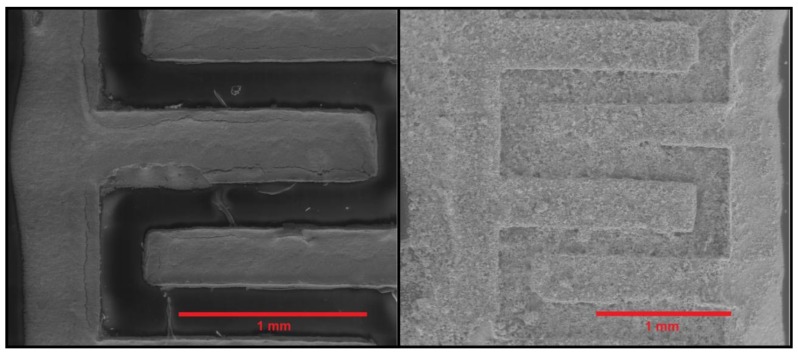
Low-magnification E-SEM images of the electrode area of a chemoresistive sensor employing a Kapton substrate. (**left**) The substrate before coating. (**right**) The same substrate after being coated with a dropped indium oxide gas sensitive film.

Considering these results, chemoresistive gas sensors were produced by depositing films of In_2_O_3_ synthesized at 500 °C. Figure 3 shows low-magnification E-SEM images of the electrode area of a Kapton transducer before and after being coated with the gas-sensitive film. The microdrop coating method employed succeeds in completely and homogeneously recovering the electrode area with the gas-sensitive indium oxide film. 

### 3.2. Gas Sensing Results

The sensors were exposed to different concentrations of NO_2_, NH_3_, and H_2_ and tested by means of DC resistance measurements. Different operating temperatures were tested to determine the optimal. Sensing experiments were carried out under dry air conditions. For all measurements, 60 min of synthetic air were employed to clean the sensor surface and stabilize its baseline signal, followed by 30 min pulses of target gas at different concentrations alternated with 30 min pulses of synthetic air to enable the sensors to recover their baseline. All these experiments were conducted with the sensors placed inside a Teflon chamber (14 cm^3^) under a constant flow of 100 mL·min^−1^, either of pure, dry air, or of a gas mixture balanced in dry air. Under these conditions, the relative humidity in the test chamber where sensors were housed remained stable at 3% (temperature was 25 °C). In an attempt to determine the optimal operating temperature for the different species measured, sensors were operated at 100 °C, 150 °C, 200 °C and 250 °C. The highest working temperature was set to 250 °C. According to the specifications of the silver ink used to draw the electrodes, it is not recommended to heat the substrates above 250 °C. At higher operating temperatures, electrodes would suffer cracks, oxidation processes, and could even detach from the substrate, which would shorten the sensor lifetime.

**Figure 4:**
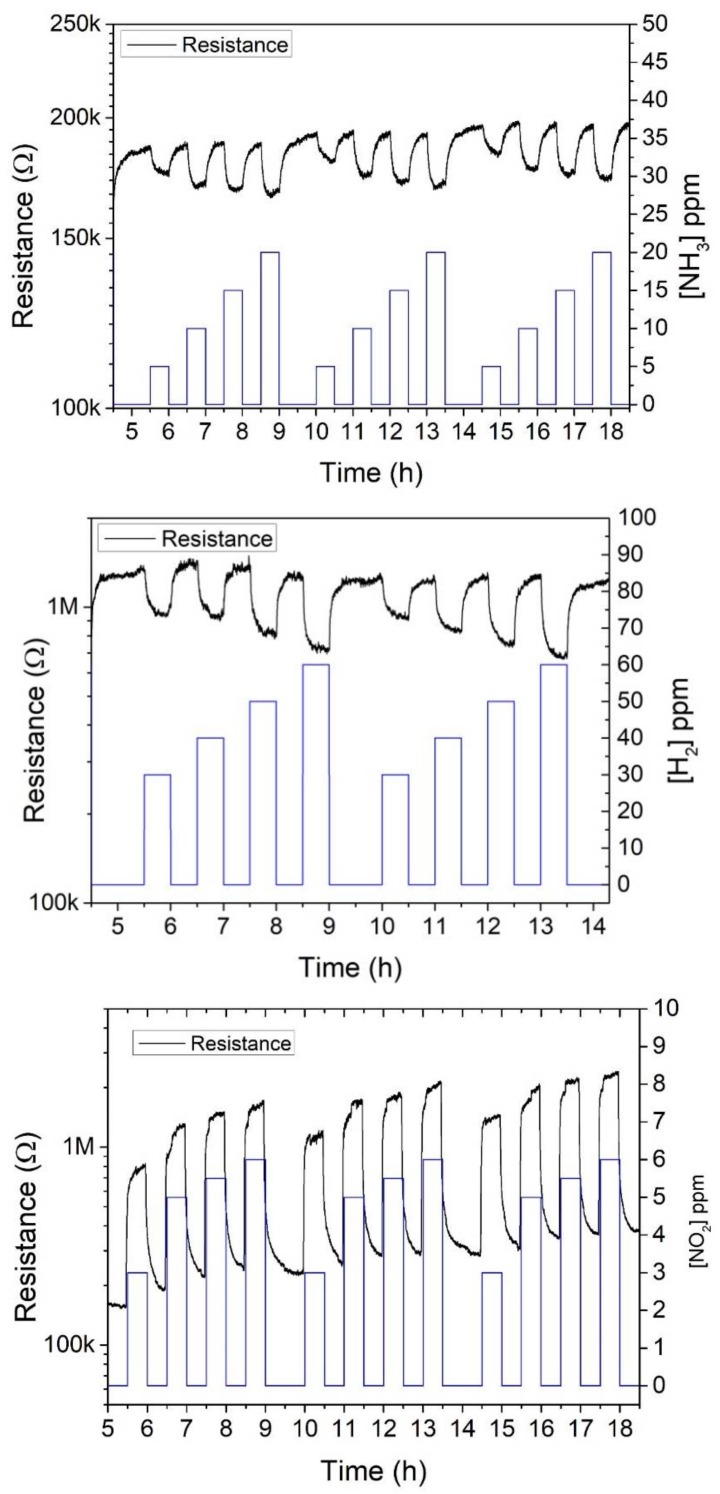
Dynamic evolution of sensor resistance for successive response and recovery pulses of the different species tested. The upper panel corresponds to NH_3_ pulses for a sensor operated at 150 °C. The middle panel corresponds to H_2_ pulses for a sensor operated at 250 °C. The lower panel corresponds to NO_2_ pulses for a sensor operated at 150 °C.

Figure 4 shows the typical dynamic evolution of the resistance of a sensor when exposed to successive pulses of increasing concentrations, of the different species tested. For each species, the response displayed was recorded at the optimal operating temperature of the sensor.

In_2_O_3_ behaves as an n-type semiconductor and sensor resistance decreases for reducing species such as NH_3_ or H_2_ and increase for oxidizing species like NO_2_. Each cycle of increasing concentration pulses is repeated at least two times for each panel in Figure 4. The responses are highly reproducible. Some baseline drift is present that is especially visible for NO_2_. This is due to the strong interaction of this oxidizing species with the gas sensitive film—which combined to the relatively low operating temperature of the sensor (i.e., 150 °C)—does not allow for a complete desorption of adsorbed species and baseline recovery during each cleaning phase [18].

#### 3.2.1. NH_3_ Results

**Figure 5:**
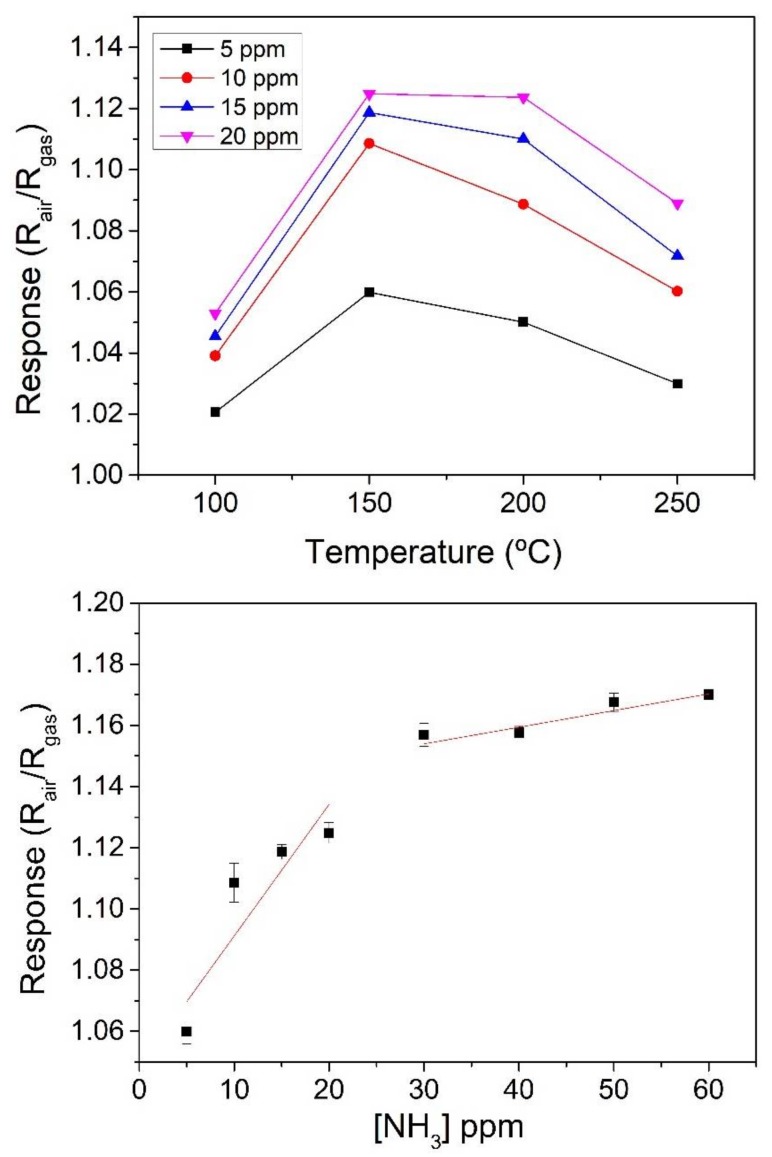
Effect of the operating temperature on the response towards different concentrations of NH_3_ (**upper panel**). Calibration curve towards NH_3_ for a sensor operated at the optimal working temperature of 150 °C (**lower panel**).

Figure 5 (upper panel) shows the effect of the operating temperature on the response to NH_3_. The response as a function of temperature curve shows the typical volcano shape for metal oxides [19]. As shown in Figure 5, the response towards NH_3_ is maximum when the sensor is operated at 150 °C. Elouali et al. [4] reported an optimum operating temperature of 450 °C to 10 ppm concentration of NH_3_ with a response of 1.76 for In_2_O_3_ prepared via continuous hydrothermal flow synthesis. In [20] an operating temperature of c.a. 175 °C was reported, with responses of 1.3 and 1.05 for 500 ppm and 100 ppm concentration of NH_3_, respectively for In_2_O_3_ thin films prepared via thermal evaporation. Our sensor showed a response of 1.12 to a 20 ppm pulse of NH_3_ comparing favorably with the optimum operating temperature for the sensors mentioned before.

The lower panel in Figure 5 shows the calibration curve for NH_3_ when the sensor is operated at 150 °C—i.e., the optimal operating temperature for this species. Measurements present low standard deviation, below 1%, even lower for the higher NH_3_ concentrations, which is indicative of high repeatability. The responsiveness towards NH_3_ of the indium oxide octahedra sensors is moderated [4,20] and shows a tendency to become saturated for concentrations higher than 30 ppm. When operated at the optimal operating temperature of 150 °C, response and recovery times (t90) for ammonia were 190 and 410 s, respectively.

#### 3.2.2. H_2_ Results

**Figure 6:**
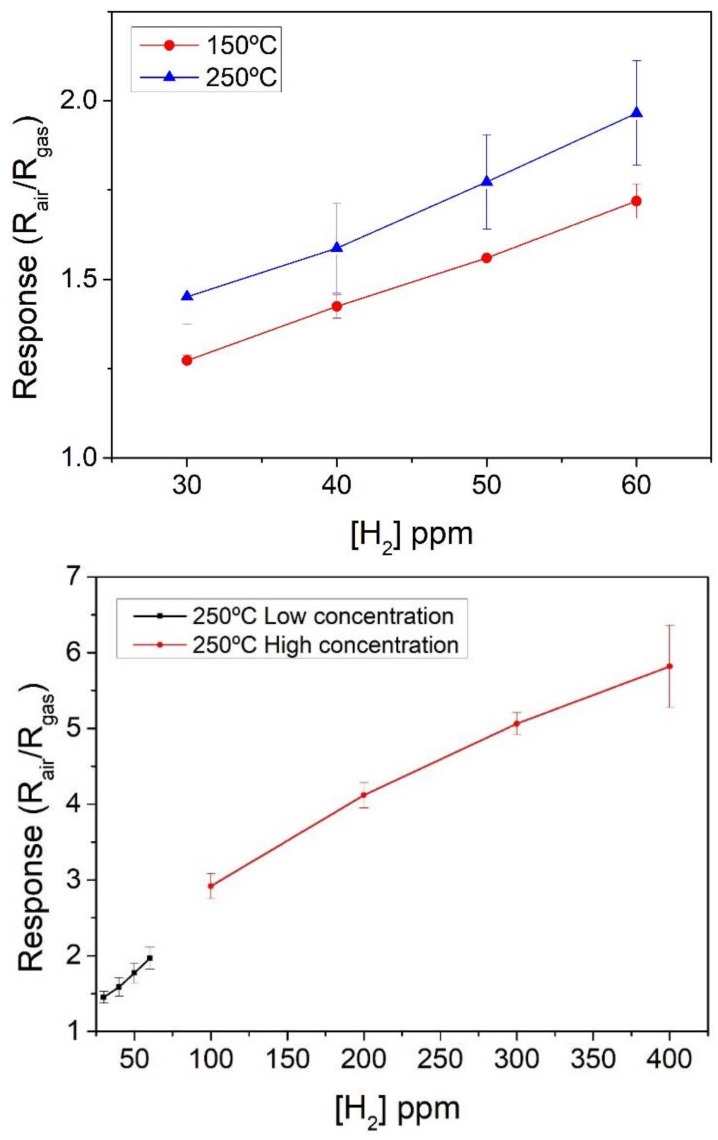
Effect of the operating temperature on the response towards different concentrations of H_2_ (**upper panel**). Calibration curve towards hydrogen for a sensor operated at the maximum allowed working temperature of 250 °C, which resulted in the higher responsiveness to hydrogen (**lower panel**).

The sensors fabricated were exposed also to different concentrations of H_2_. Following the same approach, in the first place, a temperature study was carried out in order to find the optimal working temperature for this particular gas. The results obtained are shown in the upper panel of Figure 6. The response towards H_2_ of the In_2_O_3_ sensors increased with operating temperature, therefore, the higher responses were obtained at 250 °C. Even though increasing the operating temperature of the sensors further could lead to even higher responses, the maximum temperature was set to 250 °C in order to prevent the occurrence of electrode damage, as already discussed above.

The standard deviation of H_2_ responses when the sensor was operated at 150 °C was lower than 5%. Meanwhile when the sensor was operated at 250 °C, the standard deviation of H_2_ responses increased up to 15%. The calibration curve for H_2_ shown in the lower panel of Figure 6 indicates that sensor response is quite linear for low H_2_ concentrations (up to 100 ppm). The higher concentration measured was 400 ppm of H_2_ and, up to this value, the sensor does not show response saturation. When operated at the optimal operating temperature of 250 °C, response and recovery times (t90) for hydrogen were 195 and 850 s, respectively.

Previously-reported In_2_O_3_ sensors, based on other synthesis techniques (flower-like indium oxide prepared via hydrothermal method, operated at 210 °C, [5]), shown responses magnitudes of 1.4 to H_2_ concentrations above 100 ppm. Below this concentration those sensors show no response at all, unlike the linear response reported in this work. 

#### 3.2.3. NO_2_ Results

**Figure 7:**
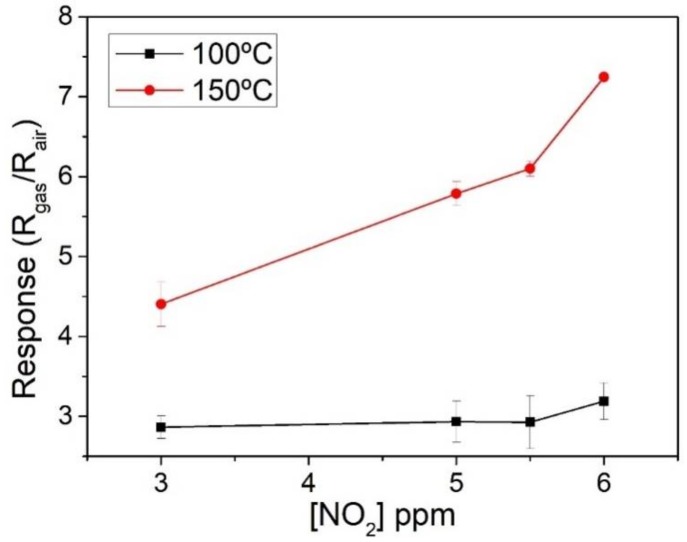
Sensor response towards NO_2_ at two different operating temperatures.

Sensors were exposed to low concentrations (3, 5, 5.5, and 6 ppm) of NO_2_ at two different temperatures; 100 °C and 150 °C. Figure 7 shows the response of the sensors at both temperatures, which is better at 150 °C. S. Roso et al. [6] reported an optimum operating temperature of 130 °C for a high-temperature grown In_2_O_3_ (octahedral nanoparticles on top of an alumina substrate), which correlates with our results. When operated at the optimal operating temperature of 150 °C, response and recovery times (t90) for nitrogen dioxide were 105 and 785 s, respectively. These are very similar to the values reported in [6].

Many indium oxide synthesis methods and sensor structures have been reported for detecting nitrogen dioxide. Indium oxide responses to nitrogen dioxide range from 352.3 (@ 100 ppb) for a sensor operating at 120 °C, as reported in [21], to the rather small one of 0.1 (@ 5 ppm) for a sensor operating at 150 °C [2]. In comparison, the response for NO_2_ (@ 5 ppm) for the sensors reported in this paper is 5.75, which is a remarkable result for such a simple and inexpensive synthesis and sensor manufacture process. In addition, this response reported here represents almost a five-fold increase in comparison to the response reported previously for indium oxide coated polyimide chemoresistors [15] in which the active film was synthesized at 400 °C. A comparison of the characteristics and gas sensing performance of different indium oxide sensors can be found in the Supplementary Materials.

## 4. Discussion

The mechanism of response to reducing species such as ammonia or hydrogen involves the interaction with oxygen species adsorbed on the surface of indium oxide. The nature of oxygen species depends on the temperature of the metal oxide [19,22,23].

O_2(g)_ ↔ O_2(ads)_

O_2(ads)_ + e^−^ ↔ O_2_^−^_(ads)_   (<150 °C)

O_2_^−^_(ads)_ + e^−^ ↔ 2O^−^_(ads)_   (150–400 °C)

O^−^_(ads)_ + e^−^ ↔ O^2−^_(ads)_   (>400 °C)

Considering that the best temperature for detecting ammonia vapors has found to be 150 °C, it can be assumed that both adsorbed molecular oxygen O_2_^−^ and O^−^ species coexist on the surface of indium oxide and, therefore, the following reactions can explain ammonia response:

4NH_3_ + 3O_2_^−^_(ads)_ ↔ 2N_2_ + 6H_2_O + 6e^−^

2NH_3_ + 3O^−^_(ads)_ ↔ N_2_ + 3H_2_O + 3e^−^

2NH_3_ + 4O^−^_(ads)_ ↔ N_2_O + 3H_2_O + 4e^−^

2NH_3_ + 5O^−^_(ads)_ ↔ 2NO + 3H_2_O + 5e^−^

The release of electrons explains the decrease in sensor resistance observed upon exposure to ammonia.

Indium oxide octahedra show response to hydrogen when operated at 250 °C. At such operating temperature, the response mechanism can be summarized as follows. Hydrogen is adsorbed and reacts with O^−^ species, resulting in the formation of water molecules, which eventually desorb from the surface of indium oxide:

H_2_ + O^−^_(ads)_ ↔ H_2_O + e^−^

This response to hydrogen could be increased further by loading the film with Pt or Pd nanoparticles [6]. Pt or Pd nanoparticles supported onto n-type metal oxides have been reported to enhance response towards hydrogen via a combination of chemical and electronic sensitization effects [19,22,23]. 

When operated at 150 °C, pure indium oxide octahedra show a remarkable response towards NO_2_ and, considering the small response measured for the other species tested at such low temperature, a certain degree of selectivity towards nitrogen dioxide is achieved. Additionally, ambient moisture facilitates the adsorption of NO_2_ on the surfaces of indium oxide octahedra [6] or of indium oxide nanoparticles [18], further increasing sensor response and sensitivity to this species.

Recently we employed diffuse reflectance infrared Fourier transform spectroscopy (DRIFTS) under operando conditions to identify the surface species involved in the sensing of nitrogen dioxide, employing indium oxide [18]. It was found that different sensing mechanisms were in place depending on the operating temperature. While the electrical response of In_2_O_3_ results from the adsorption of NO_2_, this adsorption differs substantially due the changes in the chemical nature of the surface of indium oxide at different temperatures. In particular, when the indium oxide sensor is operated at low temperatures, gas sensing involves surface hydroxyls with and without hydrogen bonds present over the surface. Hydrogen-bonded hydroxyl groups are consumed when the sensor is exposed to NO_2_. This facilitates NO_2_ adsorption, which withdraws electrons via the conduction band of In_2_O_3_ and, therefore, increases sensor resistance. In addition, adsorbed NO_2_ forms surface nitrites that interact with isolated surface hydroxyls. Surface nitrites irreversibly increase in concentration during successive exposures to NO_2_ at low operating temperatures, but this does not directly translate into a drift. In fact, sensor response is dominated by the hydrogen-bonded hydroxyl groups mediated NO_2_ adsorption mechanism. 

In the experiments reported here, even though dry air was used as carrier gas, the relative humidity inside the sensor chamber stabilized at 3% (@ 25 °C) and, given the fact that the operating temperature for detecting NO_2_ was set to 150 °C, the sensing mechanism discussed above holds.

## 5. Conclusions

Here we have introduced a straightforward process for synthesizing In_2_O_3_ octahedral nanoparticles. This process requires lower temperatures and obtains a significantly higher yield than a VPT method we reported previously. The quality of indium oxide octahedra (e.g., absence of contamination and crystallinity) is very similar to the one of indium oxide synthesized via the more involved VPT method, which makes the approach reported here very promising for the mass production of high-quality nanomaterials. The synthesized materials were microdrop coated onto flexible polyimide transducers for producing chemoresistive gas sensors. Their performance in the detection of different target gases, namely, H_2_, NH_3_, and NO_2_ was studied. Morphologies like octahedra offer advantages for gas sensing since they possess sharp edges and tips, providing more active sites and smooth surfaces exposed to their chemical environment. It was found, in agreement with our previous results, that pure indium oxide octahedra films were very sensitive towards nitrogen dioxide, provided that their operating temperature was set to moderate values (e.g., 150 °C). Indium oxide also shows potential for detecting hydrogen, however, loading of indium oxide octahedra with Pt or Pd nanoparticles seems necessary to increase response and lower the optimal operation temperature.

These results demonstrate the feasibility of the material synthesis and sensor production methods discussed here for achieving flexible gas sensors using inexpensive polymeric substrates. Flexible devices show gas sensing properties that match those of sensors onto ceramic or silicon rigid substrates. In the near future the miniaturization and optimization of the flexible transducer together with the development of a flexible smart tag for detecting nitrogen dioxide will be envisaged.

## Supplementary Materials

The following are available online at http://www.mdpi.com/1424-8220/18/4/999/s1, Figure S1. EDX from an In_2_O_3_ sample synthesized at 400 °C. Figure S2. EDX from an In_2_O_3_ sample synthesized at 500 °C sample. Figure S3. Nitrogen dioxide calibration curves for a sensor consisting of indium oxide synthesized at 400 °C coated onto a polyimide flexible transducer. Table S1. Comparison of gas-sensing performances of In_2_O_3_ gas sensors using different structures.
