# Supplementary material for: Flexible Gas Sensors Employing Octahedral Indium Oxide Films
Source: Sensors (Basel). 2018 Mar 28;18(4):999. doi: 10.3390/s18040999 (PMC5948632; doi:10.3390/s18040999)
Supplement: Supplementary file 1 [file sensors-18-00999-s001.pdf]

Supporting information for:

Flexible Sensors Based on Octahedral Indium Oxide Nanopowder for Gas Sensing Applications

Miriam Alvarado [1], Èric Navarrete[1], Alfonso Romero[1] José Luis Ramírez[1] and Eduard Llobet[1,*]

[1] MINOS-EMas, Universitat Rovira i Virgili, Tarragona, Spain
* Correspondence: eduard.llobet@urv.cat

The following figures S1 and S2 correspond to the EDX obtained for the samples grown at 400 ºC and 500 ºC. The as mentioned Cl remnant from the precursor has been found at 400 ºC by means of EDX. At 500 ºC the temperature is high enough to promote its elimination, or at least, resulting its concentration below the limit of detection for such technique.

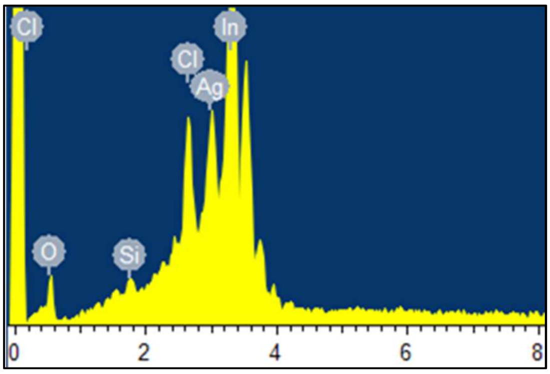

(keV)

Figure S1. EDX from an In_2O_3 sample synthesized at 400 ºC. The presence of Cl contamination is revealed. The silver (Ag) signal is due to the printed electrodes. Silicon (Si) is present due to the holder.

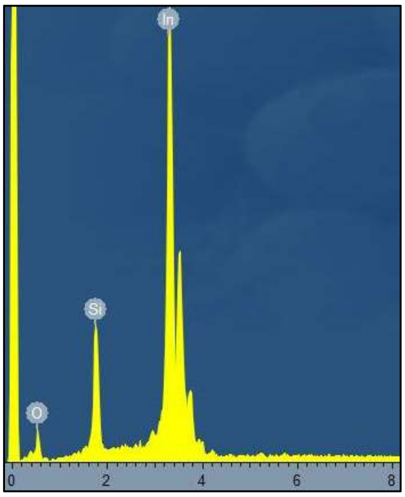

(keV)

Figure S2. EDX from an In_2O_3 sample synthesized at 500 ºC sample. Silicon (Si) signal is given due to the holder.

Figure S3 summarizes the responses towards nitrogen dioxide of a flexible sensor employing indium oxide synthesized at 400 ºC. These results were reported in [15]. The responses summarized in Figure S3 are about 5 times lower than those we report now for NO_2 (in this work). The reasons for such an increase in response may be due to:

- The elimination of contamination from the gas sensitive film (Cl is present in samples synthesized at 400 ºC and this is no longer the case for samples synthesized at 500 ºC, as shown by EDX analysis).
- Better octahedral morphology and ameliorated homogeneity of the coating.

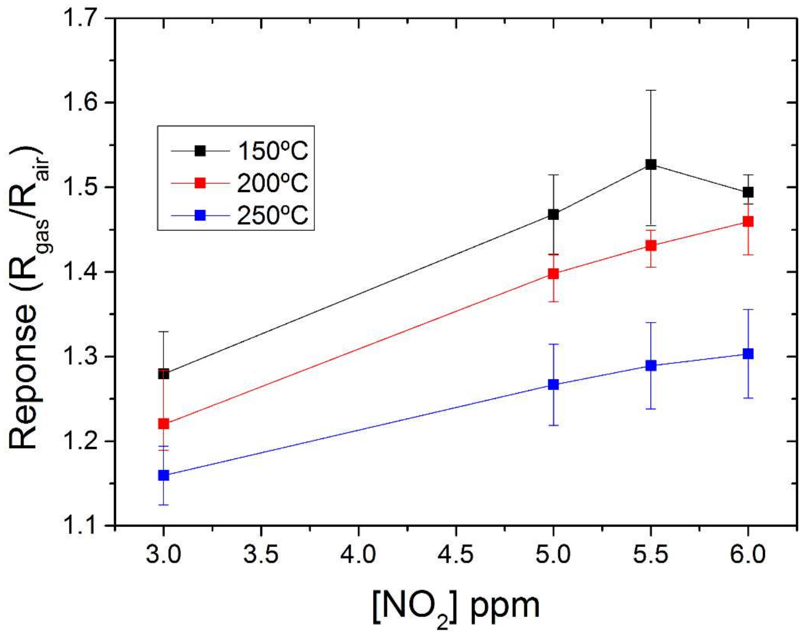

Figure S3. Nitrogen dioxide calibration curves for a sensor consisting of indium oxide synthesized at 400 ºC coated onto a polyimide flexible transducer. The different curves correspond to the different operating temperature tested. Adapted from [15].

Table S1. Comparison of gas-sensing performances of In_2O_3 gas sensors using different structures.

Sensing material	Synthesis strategy	Processing temperature/ Reaction time	Annealing temperature (Time)	Sensor fabrication	Working temperature	Response* for NO_2	Substrate	Ref
**In_2O_3 micro-cubes**	Low-temperature wet chemical	90 ºC (3 h)	400 ºC (1 h)	Mixed with distilled water	60 ºC	2.9 (500 ppb)	Ceramic tube	[S1]
**In_2O_3 nanowires**	Wet-chemical	180 ºC (30 h)	400 ºC (5 min)	Mixed with ethanol	250 ºC	2.57 (1 ppm)	Alumina tube	[S2]
**In_2O_3 microspheres**	Template-free solvent-thermal method	200 ºC (4 h)	600 ºC (3 h)	Sintered at 500ºC (2 h) Aged at 300ºC (7 days)	250 ºC	1.5 (5 ppm)	Alumina tube	[S3]
**In_2O_3 nanorod bundles**	Microwave hydrothermal	140 ºC (0,5 h)	550 ºC (2 h)	Dispersed in water, dried at RT, aged at 400 ºC (2 h)	100 ºC	87 (1 ppm)	Ceramic tube	[S4]
**In_2O_3 nanobricks**	Bath heating	96 ºC (2 h)	400 ºC (2 h)	Mixed with ethanol, dried at RT. Aged at 130 ºC (24 h)	50 ºC	402 (500 ppb)	Ceramic	[S5]
**In_2O_3 nanospheres**	Solvothermal method	100 ºC (24 h)	500 ºC (2 h)	Dispersed in deionized water	120 ºC	371.9 (1 ppm)	Ceramic tube	[S6]
**In_2O_3 nanorod clusters**	Solvothermal method	160 ºC (12 h)	500 ºC (2 h)	Mixed with deionized water, dried at RT, sintered at 500 ºC (2 h)	150 ºC	41 (500 ppb)	Alumina tube	[S7]
**In_2O_3 octahedra**	Vapor phase transport	1000 ºC (2 h)		Mixed with 1,2-propanediol	130 ºC	120 (1 ppm)	Alumina	[S8]
**In_2O_3 octahedra**	Oxidation	500 ºC (2 h)		Mixed with 1,2-propanediol, dried at 150 ºC	150 ºC	5.75 (5 ppm)	Polyimide	This work

* Response calculated $S=R_g/R_a$

Supporting information references

S1. Navale, S. T.; Liu, C.; Yang, Z.; Patil, V. B.; Cao, P.; Du, B.; Mane, R. S.; Stadler, F. J. Low-temperature wet chemical synthesis strategy of In2O3for selective detection of NO2down to ppb levels. *J. Alloys Compd.* **2018**, *735*, 2102–2110, doi:10.1016/j.jallcom.2017.11.337.

S2. Xu, P.; Cheng, Z.; Pan, Q.; Xu, J.; Xiang, Q.; Yu, W.; Chu, Y. High aspect ratio In2O3 nanowires: Synthesis, mechanism and NO2 gas-sensing properties. *Sensors Actuators, B Chem.* **2008**, *130*, 802–808, doi:10.1016/j.snb.2007.10.044.

S3. Cheng, Z.; Song, L.; Ren, X.; Zheng, Q.; Xu, J. Novel lotus root slice-like self-assembled In2O3microspheres: Synthesis and NO2-sensing properties. *Sensors Actuators, B Chem.* **2013**, *176*, 258–263, doi:10.1016/j.snb.2012.09.048.

S4. Li, X.; Yao, S.; Liu, J.; Sun, P.; Sun, Y.; Gao, Y.; Lu, G. Vitamin C-assisted synthesis and gas sensing properties of coaxial In<inf>2</inf>O<inf>3</inf> nanorod bundles. *Sensors Actuators, B Chem.* **2015**, *220*, 68–74, doi:10.1016/j.snb.2015.05.038.

S5. Han, D.; Zhai, L.; Gu, F.; Wang, Z. Highly sensitive NO2gas sensor of ppb-level detection based on In2O3nanobricks at low temperature. *Sensors Actuators, B Chem.* **2018**, *262*, 655–663, doi:10.1016/j.snb.2018.02.052.

S6. Xiao, B.; Zhao, Q.; Wang, D.; Ma, G.; Zhang, M. Facile synthesis of nanoparticle packed In_2O_3 nanospheres for highly sensitive NO_2 sensing. *New J. Chem.* **2017**, *41*, 8530–8535, doi:10.1039/C7NJ00647K.

S7. Xu, X.; Zhang, H.; He, C.; Pu, C.; Leng, Y.; Li, G.; Hou, S.; Zhu, Y.; Fu, L.; Lu, G. Synthesis and NO_2 sensing properties of indium oxide nanorod clusters via a simple solvothermal route. *RSC Adv.* **2016**, *6*, 47083–47088, doi:10.1039/C6RA01958G.

S8. Roso, S.; Bittencourt, C.; Umek, P.; González, O.; Güell, F.; Urakawa, A.; Llobet, E. Synthesis of single crystalline In_2O_3 octahedra for the selective detection of NO_2 and H_2 at trace levels. *J. Mater. Chem. C* **2016**, *4*, 9418–9427, doi:10.1039/C6TC03218D.
